# Kaempferol Protects Against Cerebral Ischemia Reperfusion Injury Through Intervening Oxidative and Inflammatory Stress Induced Apoptosis

**DOI:** 10.3389/fphar.2020.00424

**Published:** 2020-04-15

**Authors:** Jing Wang, Junqin Mao, Rong Wang, Shengnan Li, Bin Wu, Yongfang Yuan

**Affiliations:** ^1^ Department of Pharmacy, Shanghai ^9th^ People’s Hospital, Shanghai Jiao Tong University School of Medicine, Shanghai, China; ^2^ Department of Pharmacy, Shanghai Pudong New Area People’s Hospital, Shanghai, China

**Keywords:** kaempferol, cerebral ischemia reperfusion, oxidative stress, inflammation, Nrf-2, NF-κB

## Abstract

The aim of this research is to investigate the potential neuro-protective effect of kaempferol which with anti-oxidant, anti-inflammatory, and immune modulatory properties, and understand the effect of kaempferol on reducing cerebral ischemia reperfusion (I/R) injury *in vivo*. Male adult Sprague Dawley (SD) rats were pretreated with kaempferol for one week *via* gavage before cerebral I/R injury operation. We found that kaempferol treatment can reduce the cerebral infarct volume and neurological score after cerebral I/R. Rats were sacrificed after 24 h reperfusion. We observed that kaempferol improved the arrangement, distribution, and morphological structure of neurons, as well as attenuated cell apoptosis in brain tissue *via* hematoxylin and eosin (H&E) staining, Nissl staining and TUNEL staining. Superoxide dismutase (SOD), malondialdehyde (MDA), and glutathione peroxidase (GSH) kit analysis, enzyme-linked immunosorbent (ELISA) assay, real-time PCR, Western blot, and immunohistochemical examination indicated that kaempferol mitigated oxidative and inflammatory stress *via* regulating the expression of proteins, p-Akt, p-GSK-3β, nuclear factor erythroid2-related factor 2 (Nrf-2), and p-NF-κB during cerebral I/R, thus increasing the activity of SOD and GSH, meanwhile decreasing the content of MDA in serum and brain tissue, as well as restoring the expression levels of tumor necrosis factor alpha (TNF-α), interleukin-1β (IL-1β), and IL-6 *in vivo*. Taken together, this study suggested that kaempferol protects against cerebral I/R induced brain damage. The possible mechanism is related with inhibiting oxidative and inflammatory stress induced apoptosis.

## Introduction

Ischemia and hemorrhagic stroke are common cerebrovascular sicknesses, with proportion of 87 and 13%, respectively (Broussalis et al., 2012; Xue et al., 2017). A long period of ischemia results in the shortage of oxygen and glucose which destroy the cellular homeostasis. Theoretically, restoring blood flow is necessary. However, reperfusion can aggravate brain damage (Zhang et al., 2013). Cerebral ischemia reperfusion (I/R) leads to the imbalance of brain energy, which is characterized by an insufficient oxygen supply and restoration of blood flow, meanwhile involves complex and multi-factorial mechanism, and further causes cerebral injury including neuro-inflammation, neuronal damage, and cerebral edema (Zhou et al., 2017). Due to narrow therapeutic time window, the options for acute ischemia remain very limited, and few neuroprotective treatments have been successfully developed to prevent ischemic injury (Li Q. et al., 2019). I/R results in irreversible damage to brain tissue as well as subsequent disability and a high morbidity to patients. Thus, finding more safe and effective therapeutic agent is becoming more pressing (Ma et al., 2015; Yu et al., 2016). During I/R injury, the excessive production of reactive oxygen species and reactive nitrogen species, excitatory amino acid toxicity, and inflammatory reaction are implicated in the neuronal damage (Li Z. et al., 2019).

During I/R injury, oxidative stress and a mediator of the secondary injury process that involving inflammation and apoptosis are considered as crucial roles (Xie et al., 2017; Fu et al., 2018).Thus, improving the expression of the endogenous antioxidant proteins may be an effective approach to reduce cell and brain tissue injury. Research evidence indicates that there is a close relationship between endogenous antioxidant systems and nuclear factor erythroid2-related factor 2 (Nrf-2) (Danilov et al., 2009). Nrf-2 is regarded as an important transcription factor that through binding to antioxidant response elements (AREs) induces the transcription of phase II detoxifying anti-oxidant genes (Wang R. et al., 2018). Present studies demonstrate that GSK, a serine/threonine protein kinase, plays an important role in regulating and degrading Nrf-2 in a Keap1-independent manner (Rada et al., 2011). Moreover, activation of AKT stimulates phosphorylation of GSK-3β which is involved in neuroprotective effect against transient forebrain ischemia. Nuclear factor-kappa B (NF-κB) is a significant transcription factor that regulates inflammation (Qi et al., 2012). A large number of inflammatory factors, including tumor necrosis factor alpha (TNF-α), interleukin-1β (IL-1β), and IL-6, can be regulated by the activation of NF-κB (Umesalma and Sudhandiran, 2010).

Recent study showed that NF-κB is activated in cerebral vascular endothelial cells after cerebral ischemia, which triggers a dramatically increasing production of inflammatory cytokines, leading to an inflammatory cascade reaction and aggravating brain damage (Shi et al., 2014).

Kaempferol (3,4′,5,7-tetrahydroxyflavone) is one of the widest distributed flavonoids, and abundant in many kinds of traditional Chinese medicine, foods, and nutraceuticals. It has reported that kaempferol can have beneficial and/or protective effects against several diseases, due to its anti-oxidant, anti-inflammatory, and immune modulatory properties *in vitro* and *in vivo* (Wang J. et al., 2018). Previous studies exhibited that kaempferole quips with various beneficial pharmacology effects, such as alleviating gamma radiation induced injury by inhibiting oxidative stress and modulating apoptotic molecules cytochrome c, Prx-5, caspase 9, and caspase 3 expression, attenuating the anoxia/reoxygenation-induced cardiomyocyte apoptosis through SIRT1 mediated mitochondrial pathway (Guo et al., 2015) and inhibiting pancreatic cancer cell growth and migration *via* blocking EGFR-related pathway (Lee and Ki, 2016). Except that, kaempferol was reported can selectively inhibit human monoamine oxidases-A (MAO-A) in brain mitochondrial. The role of MAOs is to catalyze the α-carbon two-electron oxidation of amine substrates in the peripheral tissues and brain (Gidaro et al., 2016). So far, there are still lack of reports about the neuroprotective effect and possible mechanisms of kaempferol on I/R *in vivo*. Therefore, the present study aims to investigate whether kaempferol with neuroprotective effect and understand the potential mechanisms.

## Methods and Materials

### Animals and Reagents

Male adult Sprague Dawley (SD) rats (body weight, 240–260g) were all purchased from Changzhou Cavens Experimental Animal Co. (Jiangsu, China). All rats were managed under specific-pathogen-free (SPF) conditions and used according to the Guidelines of National Institutes of Health on the Care and Use of Laboratory Animals. This research was approved by the Scientific Investigation Board of the Second Military Medical University (Number: SYXK2017-0004). Kaempferol (purity≥98%) was purchased from Dalian Meilun Biology Technology Co. (Dalian, China). Kaempferol was diluted with 0.5% sodium carboxymethylcellulose (CMC-Na) to different concentrations. 2,3,5-triphenyltetrazolium chloride (TTC) was purchased from Sigma-Aldrich Co. (St. Louis, MO, USA); The superoxide dismutase (SOD), malondialdehyde (MDA), and glutathione peroxidase (GSH) assay kit were purchased from Nanjing Jiancheng Bioengineering Institute (Nanjing, China).

### Experimental Group and Drug Administration

Firstly, in order to observe the protective effect of kaempferol in rat I/R injury, rats were randomly divided into five groups and drug was administrated continuously one week before operation: Sham group (5 rats; 0.5% CMC-Na; intragastrically administrated; dosing one time per day for one week; expose left common, external and internal artery, but no blockage), I/R group (5 rats; 0.5% CMC-Na; intragastrically administrated; dosing one time per day for one week; ischemia for 2 h and reperfusion for 24 h), three Kaempferol treatment groups (each group 5 rats; 1.75, 3.49, 6.99 mM, respectively; intragastrically administrated; 1 ml/kg weight; dosing one time per day for one week; ischemia for 2 h and reperfusion for 24h).

Secondly, to understand kaempferol neuroprotective effect, rats were randomly divided into three groups and 10 rats in each groups: Sham group, I/R group and 6.99 mM kaempferol treatment group. The 6.99 mM kaempferol treatment group was intragastrically administrated 1 ml/kg weight, continuous one week, prior to ischemia 2 h and reperfusion 24 h operation. The Sham group and I/R group were administrated with equal volume of 0.5% CMC-Na solution. After operation, there was one rat mortality in 6.99 mM kaempferol treatment group. 5 rats brain tissues in sham group and I/R group, 4 rats brain tissues in kaempferol treatment group were fixed with 4% formaldehyde, meanwhile 5 rats brain tissues in each groups were homogenized. Whole blood samples were collected from rat aortaventralis without anticoagulant except mortality rat. H&E stain, Nissl stain, TUNEL stain, and immunohistochemical examination were share the same tissue samples which were fixed.

### Cerebral I/R Model and Neurological Score

I/R rat model was operated according to Longa’s methods with minor revisable (Bösel et al., 2005). Rats were anesthetized with 4% isoflurane (4% for anesthesia induction; 2% for anesthesia maintenance, 3 ml/kg). Then carefully exposed the left common carotid artery (CCA), internal carotid artery (ICA), and external carotid artery (ECA). The left CCA and the ECA were blocked with micro-vascular aneurysm clips, followed by occluding the middle cerebral artery (MCA) by inserting a nylon filament (Product No:2634-100; Beijing Sunbio Biotech, Beijing, China) coated through the ICA. Finally, the filament was slowed removed from ICA to achieve reperfusion 2 h after occlusion. Then rats continued to be monitored in the same SPF conditions. Rats in Sham group received the same procedures except filament inserted to the MCA. Neurological score was tested when rats revived after 2 h ischemia and removed filament. 1–3 score considered successful operation and included in the following experiment.

After 24 h reperfusion, the neurological score of each rat was evaluated by Longa’s method (Longa et al., 1989) of a 5-point scale: 0, no neurological deficit; 1, the contralateral torso and forelimb may not be thoroughly stretched; 2, when the tail was held, the animal may be turned to the ipsilateral side; 3, no spontaneous motor activity or falling to the left; 4, unable to walk or loss of consciousness. Researcher was blinded to the different treatments.

### Assessment of Cerebral Infarct Volume

Followed the neurological score evaluation, the rats were sacrificed, and the brains were carefully collected, sliced into five coronal sections, with each 2 mm thick. The slices were placed in 2% TTC solution, and incubated at room temperature for 15 min. Then the brain slices were fixed in 4% formaldehyde at 4°C for 24 h. Slices images were captured. The infarct volumes were calculated *via* image analysis software (Image-Pro Plus, Version 6.0). Normal brain section was stained to red and the infarct section was stained to white. The infarct volume percentage was measured by the following equation:

Infarct volume (%) = [(normal hemisphere volume − non-infarct volume of the infarct side)/normal hemisphere volume] × 100%

### Biochemical Parameters Analysis

Rats were anesthetized after 24 h reperfusion, and whole blood samples were collected from rat aortaventralis without anticoagulant. Whole blood samples were stored at room temperature for 1 h, then centrifuged at 1,000×g for 30 min. Brain tissues were dissected, penumbra to the ischemia core area was collected (**Figure S3**), and 10% tissue homogenates were prepared. The supernatant was used to determine the level of SOD, MDA, and GSH, according to the protocols provided by the manufacturer (Nanjing Jiancheng Bioengineering Institute, Nanjing, China).

### Enzyme-Linked Immunosorbent Assay (ELISA)

Rats were anesthetized, whole blood sample was obtained from rat aortaventralis, then centrifuged at 1,000×g for 30min. After centrifugal operation, serum supernatant samples were collected. Meanwhile, brain tissues were dissected and homogenized. The expression levels of TNF-α, IL-1β, and IL-6 in the serum and brain tissue were determined according to TNF-α, IL-1β, and IL-6 ELISA kit (R&D Systems, Minneapolis, USA) instructions, respectively.

### Real-Time PCR Assay

Brain tissues were dissected and homogenized using a TL2010 grinding instrument (DHS Life Science& Technology, Beijing, China). Total RNA was extracted using the TRIzol reagent (Invitrogen, Carlsbad, CA, USA) and determined the concentration and purity through Nanodrop 2000 spectrophotometer (Thermo Fisher Scientific, Gene Company Limited, Shanghai, China). Then, the total RNA was reverse transcribed by the PrimeScript™ RT Master Mix reagent kit (Takara, Shiga, Japan). TNF-α, IL-1β, IL-6, and GAPDH mRNA expression level were detected by the SYBR Premix Ex Taq™ kit (Takara, Japan). cDNA was amplified using a three-step program. Ct values were used to calculate the mRNA expression level. The primers were synthesized by Sangon Biotech Co. Ltd (Shanghai, China) and are listed in **Table 1**.

**Table 1:** Gene primers sequences for mRNA amplification.

Gene name	Forward(5′-3′)	Reverse(5′-3′)	Product length
TNF-α	TCAGCCTCTTCTCATTCCTGC	TTGGTGGTTTGCTACGACGTG	179
IL-1β	CAGCAATGGTCGGGAC	TAGGTAAGTGGTTGCCT	118
IL-6	CCGGAGAGGAGACTTCCAGA	GGTCTGGGCCATAGAACTGA	232
GAPDH	CCAGCCCAGCAAGGATACTG	GGTATTCGAGAAGGGAGGGC	256

### Histopathological and Immunohistochemical Examination

The rats were sacrificed after 24 h reperfusion, and brain tissues were fixed with 4% formaldehyde. The brain tissues were dehydrated with different concentration gradients alcohol and embedded in paraffin and cut into 5 mm sections. To detect morphological changes in neurons, the sections were stained with hematoxylin and eosin (H&E) according to the standard procedure, and subjected to Nissl staining using 0.1% cresyl violet acetate. The number of intact cells in the penumbra of the ischemic cortex by Nissl staining was counted through five lesion regions randomly.

The sections were used for immunohistochemical examination. Briefly, these slices were dewaxed, dehydrated, and operated antigen retrieval. Furthermore, slices were blocked by 0.1% bovine serum albumin in PBS for 30 min; NF-κB and Nrf-2 primary antibodies (Cell Signaling Technology) were used to incubate slices at 4°C overnight. The goat anti-rabbit IgG (Cell Signaling Technology) was used as the secondary antibody. Afterwards, positive areas were checked with a light microscope (Olympus, Tokyo, Japan) and analyzed using Image J software.

### TUNEL Assay

To test the DNA fragmentation associated with apoptosis, the TUNEL assay was performed. The brain slices in each group were prepared and In situ Cell Death Detecting Kit (Roche Diagnostics FmbH, Penzberg, Germany) was used for achieving TUNEL staining. TUNEL staining was performed according to the routine method of kit. Apoptotic cells were identified as those with a brown-stained nucleus. Cell counting was using five randomly selected fields, and the apoptosis index was calculated as the percentage of positive cells to total cells. Samples were analyzed under a microscope and researchers were blinded to different groups.

### Western Blot Assay

The brain tissues were dissected after I/R model. The total protein was extracted from fresh brain tissues on the ice and appropriate volumes of Protein Extraction Regent (Pierce, Rockford, USA) were added. Bradford protein assay was used to measure the concentration of total protein and bicinchoninic acid (BCA) protein assay kit was performed. Western blot was performed to separate and analyze the expression levels of several target proteins. Subsequently, the membranes were incubated at 4°C overnight with corresponding primary antibodies, Akt (1:500), p-Akt (Ser473, 1:250), GSK-3β (1:500), p-GSK-3β (Ser9, 1:250), Nrf-2 (1:500), p-NF-κB (Ser536, 1:100), and GAPDH (1:1,000) (Cell signaling technology. Co) included. Then, membranes were washed with Tris-buffered saline with 0.05% Tween-20 (TBST) for 5 min × 3 times. Followed incubating appropriate secondary antibodies (Kangchen, Shanghai, China) at room temperature respectively, and detected with chemiluminescence plus detection system (Fusion FX7 Spectra; VilberLourmat, Eberhardzell, Germany). The intensities of each protein bands were measured with Quantity One software.

### Statistical Analysis

All the results are shown as the Mean ± SEM; the differences among multiple independent comparisons were analyzed by one-way analysis of variance test, followed by Student-Newman-Keuls *post hoc* tect. Nonparametric test followed by Kruskal-Wallis H test was used to analyze the results of neurological score. *P ≤* 0.05 was considered statistically significant differences.

## Results

### Kaempferol Ameliorated Neurological Scores and Reduced Infarct Volume

**Figure 1:**
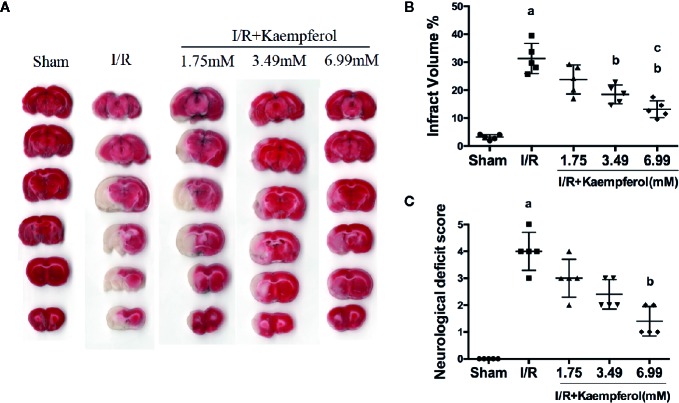
Kaempferol ameliorated neurological scores and infarct volume. Rats were administrated with 1.75mM, 3.49mM and 6.99mM kaempferol for 7 days before I/R operation. Infarct volumes were assessed by TTC staining. **(A)** Representative TTC staining results of brain slices in different groups. **(B)** Quantitative analysis of brain infarct volumes. **(C)** Neurological scores in different groups. n=5, *^a^p* < 0.05, compared with the sham group; *^b^p* < 0.05, compared with the I/R model group; *^c^p* < 0.05, compared with the I/R+kaempferol1.75mM group.

To confirm the protective effect of kaempferol on cerebral I/R injury, cerebral infarct areas and neurological scores in each group were evaluated by TTC staining and Longa’s way, respectively. Firstly, we confirmed that the CMC-Na solution didn't interfere the results of MCAO operation (**Figure S2**). From **Figures 1A, B**, we can find infarct volume in I/R group compared to Sham group (*P* < 0.05, Sham group *vs.* I/R group). The cerebral infarct area in Kaempferol treatment groups (1.75, 3.49, 6.99 mM) are all smaller than I/R group, especially Kaempferol 3.49 and 6.99 mM treatment groups (*P* < 0.05, *P* < 0.05; Kaempferol 3.49 mM treatment group *vs.* I/R group, Kaempferol 6.99 mM treatment group *vs.* I/R group). Meanwhile, infarct volume in kaempferol 6.99 mM treatment group is smaller than kaempferol 1.75 mM treatment group (*P* < 0.05, Kaempferol 6.99 mM treatment group *vs.* Kaempferol 1.75 mM treatment group). Kaempferol also can increase cell viabilities in vitro after oxygen and glucose deprivation (**Figure S1**). From **Figure 1C**, rats subjected to I/R showed apparent severe neurological deficits (*P* < 0.05, I/R group *vs.* Sham group). Meanwhile, Kaempferol treatment groups (1.75, 3.49, 6.99 mM) can reduce neurological deficit scores compared with I/R group, especially kaempferol 6.99 mM treatment group (*P* < 0.05; Kaempferol 6.99 mM treatment group *vs.* I/R group).

### Kaempferol Protected Against I/R-Induced Brain Tissue Injury and Weakened the Brain Tissue Apoptosis

**Figure 2:**
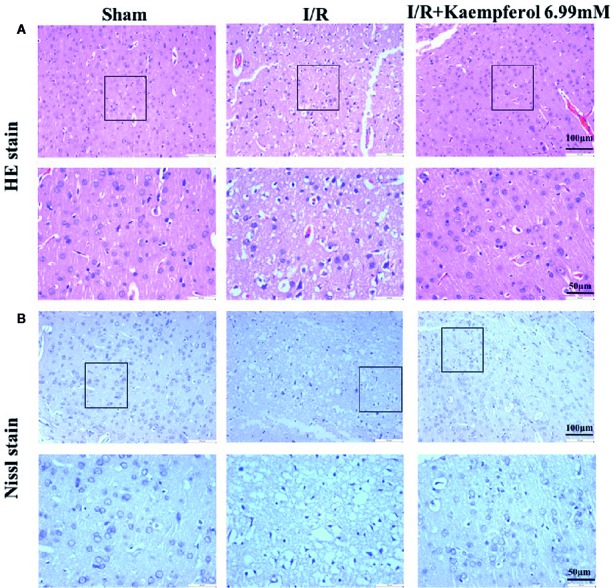
Kaempferol inhibited I/R-induced brain tissues injury. **(A)** H&E staining results. **(B)** Nissl staining results. n=4, Original magnification 200× and 400×, respectively. The area in box was presented as magnification 400x.

**Figure 3:**
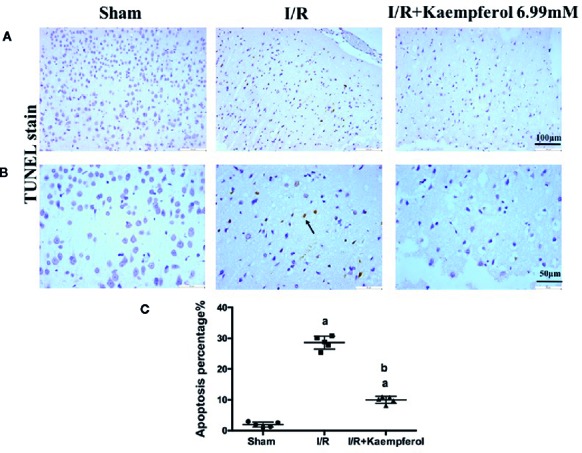
Kaempferol protected against I/R-induced neurons apoptosis in brain tissues. TUNEL staining results were showed in original magnification 200× **(A)** and 400× **(B)**, respectively. Quantification of TUNEL-positive cells in cerebral cortex **(C)**. n=4, *^a^p* < 0.05, compared with the sham group; *^b^p* < 0.05, compared with the I/R model group. Black arrow points the apoptotic cell.

We thus observed the protective effects of kaempferol on cell injury in the rat brain tissues after I/R injury. We used H&E staining to check the morphological changes. As shown in **Figure 2A**, in the cerebral cortex, the cells with abundant cytoplasm and clear nuclei were all arranged orderly in the Sham group. However, most neurons in the ischemic penumbra of cerebral cortex presented shrunken and deep stained in I/R group. Contrary to I/R group, residual neuron structures were improved in the kaempferol treatment group. Visible membranes and nuclei and more intact neurons were observed in the kaempferol treatment group. Nissl staining (**Figure 2B**) indicated that the large number of cells were shrunk with an enlarged intercellular space and more dark color staining in the I/R group relative to Sham group. Meanwhile, these characteristic changes were all improved in kaempferol treatment group. Furthermore, to determine the effect of kaempferol on neurons apoptosis TUNEL staining was used. **Figures 3A–C** demonstrated that kaempferol can significantly ameliorated I/R induced neurons apoptosis (*P* < 0.05, I/R group *vs.* Kaempferol treatment group). These results suggested that kaempferol relieves the damage caused by cerebral I/R

### Effect of Kaempferol on the Expression Levels of SOD, MDA, and GSH in the Serum and Brain Tissue

**Figure 4:**
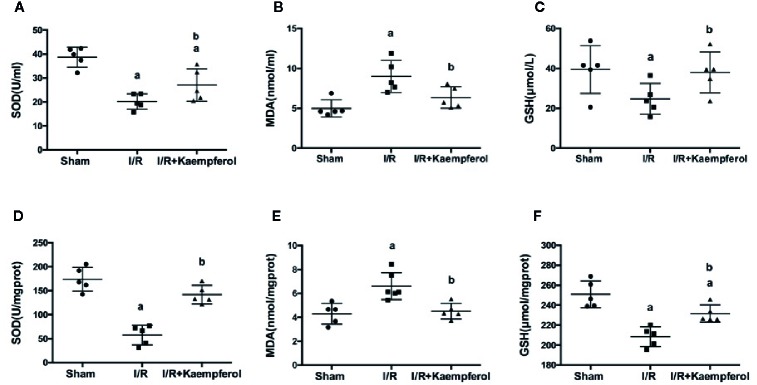
Effect of kaempferol on the expression levels of SOD **(A, D)**, MDA **(B, E)** and GSH **(C, F)** in serum (n=9) and brain tissues (n=5) after ischemia/reperfusion injury. *^a^p* < 0.05, compared with the sham group; *^b^p* < 0.05, compared with the I/R model group.

To understand the protective effect of kaempferol on cerebral I/R, the activity level of SOD, MDA, and the content of GSH in the serum and brain tissues were determined. We observed that, compared with Sham group, the activities of SOD and GSH in serum and brain tissues were significantly decreased in I/R group (*P* < 0.05; *P* < 0.05; *P* < 0.05; *P* < 0.05; Sham group *vs.* I/R group). At the same time the content of MDA in serum and brain tissue were increased in I/R group (*P* < 0.05; *P* < 0.05; Sham group *vs.* I/R group). Surprisingly, kaempferol treatment not only significantly increased the activities of SOD (**Figures 4A, D**; *P* < 0.05; *P* < 0.05; Kaempferol 6.99 mM treatment group *vs.* I/R group) and GSH (**Figures 4C, F**; *P* < 0.05; *P* < 0.05; Kaempferol 6.99 mM treatment group *vs.* I/R group), but also the content of MDA was decreased (**Figures 4B, E**; *P* < 0.05; *P* < 0.05; Kaempferol 6.99 mM treatment group *vs.* I/R group) relative to I/R group in serum and brain tissues. According to this results we believed that the beneficial effects of kaempferol administration under cerebral I/R are associated with anti-oxidative function.

### Effect of Kaempferol on the Expression of Inflammatory Factors TNF-α, IL-1β, and IL-6

**Figure 5:**
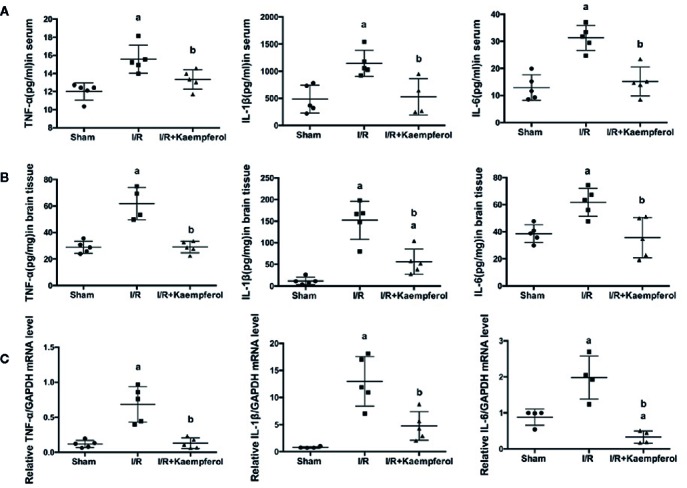
ELISA assay was performed to detect the effect of kaempferol on inflammatory factors (TNF-α, IL-1β and IL-6) expression level in serum (n=5) **(A)** and brain tissues (n=5) **(B)** after ischemia/reperfusion injury. TNF-α, IL-1β and IL-6 mRNA expression levels in brain tissues were tested by real-time PCR **(C)**. *^a^p* < 0.05, compared with the sham group; *^b^p* < 0.05, compared with the I/R model group.

To detect the expression level of inflammatory factors TNF-α, IL-1β, and IL-6 after kaempferol treatment under cerebral I/R, ELISA and real-time PCR assay were used. **Figure 5A** indicates that I/R resulted in the high expression level of TNF-α, IL-1β, and IL-6 (*P* < 0.05; *P* < 0.05; *P* < 0.05; I/R group *vs.* Sham group) in serum compared with Sham group. However, the expression levels of TNF-α, IL-1β, and IL-6 in serum showed a significant decrease after kaempferol treatment (*P* < 0.05; *P* < 0.05; *P* < 0.05; I/R group *vs.* Kaempferol treatment group). In **Figure 5B**, we found that after I/R injury the expression levels of TNF-α, IL-1β, and IL-6 in brain tissues are all significantly increased (*P* < 0.05; *P* < 0.05; *P* < 0.05; I/R group *vs.* Sham group). Kaempferol treatment can decreased TNF-α, IL-1β, and IL-6 expression levels in brain tissue (*P* < 0.05; *P* < 0.05; *P* < 0.05; I/R group *vs.* Kaempferol treatment group). Further analysis is shown in **Figure 5C**, we found that the mRNA level of TNF-α, IL-1β, and IL-6 in brain tissues were all markedly increased after I/R (*P* < 0.05; *P* < 0.05; *P* < 0.05; I/R group *vs.* Sham group). These inflammatory factors’ mRNA expression levels were remarkably down-regulated after kaempferol treatment (*P* < 0.05; *P* < 0.05; *P* < 0.05; I/R group *vs.* Kaempferol treatment group). According to the above description, we assumed that kaempferol administration under cerebral I/R are associated with anti-inflammatory effect.

### Kaempferol Regulated the Expression Levels of Akt, p-Akt, p-GSK-3β, Nrf-2, NF-κB, and p-NF-κB in Rat Brain Tissues

**Figure 6:**
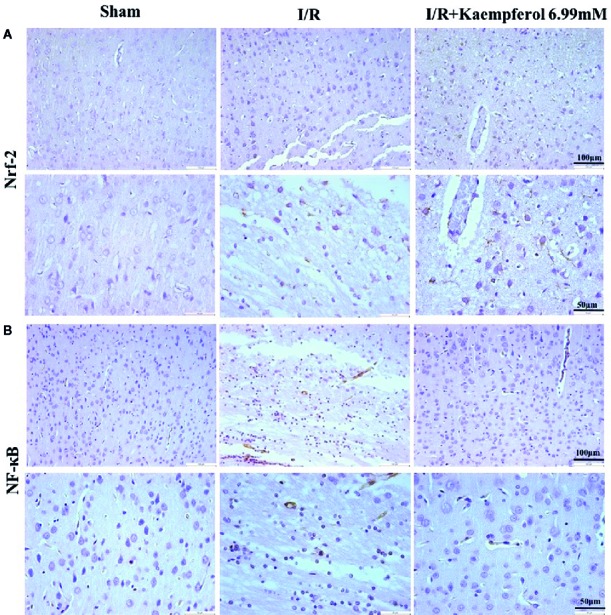
Kaempferol affected expression levels of Nrf-2 and NF-κB in cerebral cortex after ischemia and reperfusion injury. Immunohistochemical staining was used to examine the expression of Nrf-2 **(A)** and NF-κB **(B)**. n=4, Original magnification 200× and 400×.

**Figure 7:**
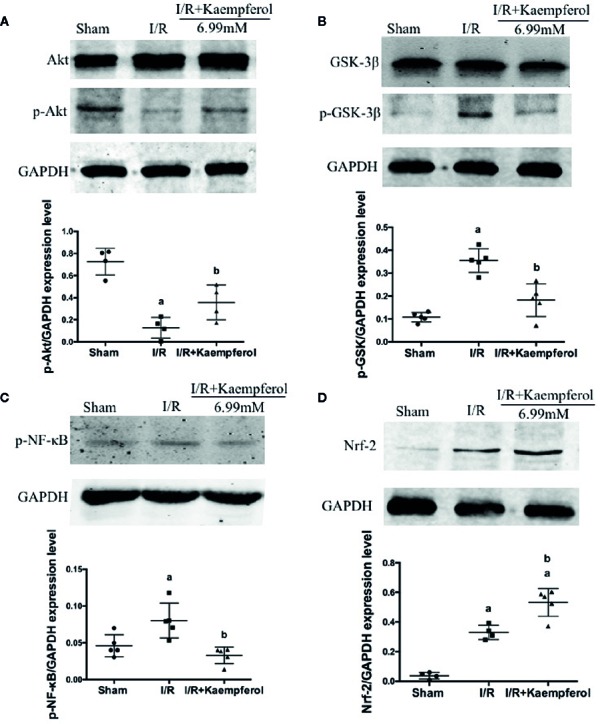
Kaempferol affected expression levels of proteins Akt, p-Akt, GSK-3β, p-GSK-3β, p-NF-κB and Nrf-2 in brain tissues after ischemia reperfusion injury. Western blot assay and Quantity One software were used to investigate and calculate the expression levels of proteins Akt and p-Akt **(A)**, GSK-3β and p-GSK-3β **(B)**, p-NF-κB **(C)** and Nrf-2** (D)**. n=5, *^a^p* < 0.05, compared with the sham group; *^b^p* < 0.05, compared with the I/R model group.

To observe the effect of kaempferol on protecting the neurons after I/R and according to the previous research results described above, the expression levels of Akt, p-Akt, p-GSK-3β, Nrf-2, NF-κB, and p-NF-κB in rat brain tissues were performed *via* western blot and immunohistochemical staining. We observed that the I/R injury increased expression of proteins NF-κB and p-NF-κB (**Figures 6B**, **7C**), p-GSK-3β (**Figure 7B**), and Nrf-2 (**Figures 6A**, **7D**), and decreased expression of protein p-Akt (**Figure 7A**) compared with Sham group. What is surprising is that the expression levels of proteins NF-κB, p-NF-κB, and p-GSK-3β were down-regulated after kaempferol treatment, and the expression levels of proteins Nrf-2, p-Akt were up-regulated in comparison with I/R group.

## Discussion

Cerebral ischemia with high morbidity and mortality, however, the complicated pathological mechanisms lead to identify a novel neuroprotective pharmacological drug becoming difficult. Accumulated evidences indicate that numerous factors are included in ischemia brain injury, involving apoptosis, excitotoxicity, oxidative stress, per-infarct depolarization, nutritive stress, reactive oxygen species (ROS), and inflammation (Xu et al., 2016; Zerna et al., 2016; Xing et al., 2018). Moreover, during the ischemia damage stage, the cell metabolism abnormalities may be resulted from the shortage of blood, oxygen, and glucose supply, which commonly led to neuronal death or apoptosis. Thus, anti-oxidant and anti-inflammation treatment strategies are being developed to treat cerebral I/R injury. Traditional Chinese medicine with characteristics of multi-function and multi-mechanism. A previous report showed kaempferol with anti-oxidant and anti-inflammation properties *in vivo* and *in vitro* because of its phenolic and hydroxyl group structure (Wang et al., 2018). Due to the complex pathological process of I/R, it is promising that kaempferol could play a powerful anti-oxidant and anti- inflammation role in brain tissue during cerebral I/R.

Firstly, detecting the cerebral infarct volume in brain tissue by TTC staining to observe the protective effect of kaempferol on cerebral I/R injury (Li et al., 2019).The results showed that kaempferol can significantly decrease the cerebral infarct volumes after I/R injury in rat model. Besides, kaempferol treatment greatly improved neurobehavioral defects. In our experiment, the rat brain tissues were detected by H&E staining and Nissl staining. The results, both in H&E stain and Nissl stain, indicated that kaempferol treatment improved the arrangement, distribution, and morphological structure of neurons. These results suggested that kaempferol relieves the damage caused by cerebral I/R.

The mechanisms of brain injury during cerebral I/R are comprehensive. ROS are believed to be a key factor of nerve damage post cerebral I/R (Granger and Kvietys, 2015). During ischemia physiological condition, few free radicals are present; thus, the absorbance of oxygen radicals decreases (Valko et al., 2007). Afterwards, on the stage of recovery, known as reperfusion, the blood supply of the tissue triggers the “explosion” of oxygen free radicals; therefore, the accumulated ROS attacks the cells and lead to injury (Droge, 2002). Following, the antioxidant enzymes, such as SOD, GSH are involved, in the defensive system for protecting against oxidative stress. MDA is an oxidative stress marker, which is also a product of lipid peroxidation reaction. These play an important role in neurons against ROS-induced cell injury (Ren et al., 2017). Hence, the anti-oxidative activities of kaempferol on neuroprotective role were investigated. Increasing SOD and GSH activities and decreasing MDA content have been observed after kaempferol treatment. Additionally, an inflammatory response develops within several hours after damage and is characterized by the activation of pro-inflammatory cytokines and infiltration of neutrophils (Fu et al., 2018). The inflammatory response makes neurological injury in a worse situation and promotes neural cells apoptosis (Anusha et al., 2017). A large number of evidences showed that the pro-inflammatory cytokines released in the periphery and neuronal apoptosis induced by cerebral I/R may occur potential injury to the hippocampal cells, which are associated with memory and learning functions (Chen et al., 2015). TNF-α, IL-1β, and IL-6 are reported as major mediators in several central nervous systems, and increased level of them related to pathological mechanisms of secondary damage, including neuronal cells apoptosis or death (Fan et al., 2014). In this study, we observed that kaempferol treatment can decreased the expression levels of TNF-α, IL-1β, and IL-6 in blood and brain tissue, meanwhile a reduction of neurons apoptosis was presented by TUNEL stain assay. According to the above description we believed that the beneficial effects of kaempferol administration under cerebral I/R are associated with anti-oxidative and anti-inflammatory, as well as reduction of neural cells apoptosis.

There are various signaling pathways involved in the cerebral I/R injury to regulate pathological mechanisms (Kisoh et al., 2019). Nrf-2 is a transcription factor implicated in mediating protection against electrophiles and oxidants and enhances cell survival in many tissues, including brain tissue (Baird and Dinkova-Kostova, 2011). Nrf-2 binds to the AREs and stimulates transcription of antioxidant proteins, which associated with scavenging ROS and glutathione (GSH) biosynthesis and regeneration (Motohashi and Yamamoto, 2004). NF-κB, a transcription factor, is a key regulator of many genes (such as TNF-α and IL-6) involved in inflammation. Activation of NF-κB stimulated by ROS and several inflammatory mediators, leads to neuron death and irreversible brain damage in cerebral I/R (Ridder and Schwaninger, 2009). Additionally, it has been reported that Nrf-2 is involved in the inflammatory response and can inhibit the activation of NF-κB (Lee et al., 2012). To assess the effect of kaempferol on molecular changes undergo cerebral I/R, we evaluated the proteins expression of Nrf-2 and NF-κB in brain tissues. We found that kaempferol treatment significantly increased the Nrf-2 expression and inhibited NF-κB expression.

Recent reports indicated that GSK-3β is a negative regulator of Nrf-2 transcription activity and participates in the distribution of Nrf-2 inside or outside of the nucleus (Chowdhry et al., 2013). GSK-3 is a component in the glycogen metabolism pathway and a crucial regulator in multiple intracellular contexts. GSK-3β is famous to be involved in a number of human disorders, including diabetes, cancer, oxidative stress, and Alzheimer’s disease at all (Juhaszova et al., 2009). GSK-3α and GSK-3β are two members of GSK-3 family, which are all predominantly expressed in the brain (Kim and Snider, 2011). Numerous reports have declared that GSK-3β, involved in the regulation of differentiation, survival, activation, or over-expression are related to ischemia neuronal death during transient cerebral ischemia (Chen et al., 2017). GSK-3β acts in memory function and cerebral ischemia-induced neurogenesis (Kim et al., 2015) depending on the site of phosphorylation. Moreover, GSK-3β is an important downstream protein in the Phosphoinositide-3-kinase/Akt (PI3K/Akt) pathway and activated Akt stimulates phosphorylation of GSK-3β. It is known that PI3K/Akt signal pathway can promote cell growth and survival in response to extracellular stimulations (Wang et al., 2010). This pathway is associated with neuroprotective function against ischemia damage (Chen et al., 2017).

To further understand protective effect of kaempferol on I/R injury, the proteins expression level of p-GSK-3β, Akt, p-Akt in brain tissues after cerebral I/R injury were tested. Surprisingly, kaempferol treatment increased the expression of p-Akt and decreased the expression of p-GSK-3β. Based on previous results and findings, we found that kaempferol with neuroprotective effect under cerebral I/R injury. The neuroprotective effect is related to anti-oxidative and anti-inflammation stresses, as well as alleviation of neurons apoptosis, which are potentially associated with up regulation of p-Akt and Nrf-2, meanwhile down regulation of p-NF-κB and p-GSK-3β. This research still exists limitations, which including we need more experiments to verify detailed molecular mechanism and modify available pharmaceutical form to further investigate kaempferol treatment effect on I/R except prophylaxis. Even though Kaempferol had a benefit on oxidative stress, inflammation, and apoptosis, it is not known which of these pathological events is more important for kaemperfol’s benefit.

## Conclusion

In conclusion, the present study confirms the neuroprotective effect of kaempferol on cerebral ischemia and reperfusion injury *in vivo*. Kaempferol protects against cerebral I/R-induced oxidative stress, inflammation, and apoptosis, which are potentially associated with up regulation of p-Akt and Nrf-2, meanwhile down regulation of p-NF-κB and p-GSK-3β.

## Data Availability Statement

The datasets analyzed in this article are publicly available. Requests to access the datasets also can be directed to wangjing93wj@126.com.

## Ethics Statement

The animal study was reviewed and approved by the Animal Ethics Committee of Shanghai 9th People’s Hospital, Shanghai Jiao Tong University School of Medicine.

## Author Contributions

JW offered substantial contributions to the conception, design of the work, and drafted the work. JM, RW, SL, and BW analyzed data for the work. YY revised manuscript critically for important intellectual content and provided approval for publication of the content.

## Funding

This project was financially supported by the National Natural Science Foundation of China (No.81803815 and No.81703779). The work was funded by the Clinical Pharmacy Innovation Research Institute of Shanghai Jiao Tong University School of Medicine, the project number are CXYJY2019MS002 and CXYJY2019QN002.

## Conflict of Interest

The authors declare that the research was conducted in the absence of any commercial or financial relationships that could be construed as a potential conflict of interest.

## Supplementary Material

Figure S1 Kaempferol increased the cell viability under OGD/R. *^##^P* < 0.01, Con group vs. OGD/R group; *^**^P* < 0.01, OGD/R group vs. OGD/R + Kaempferol 10μM treatment group.

Figure S2 The infract volume in I/R only group and I/R+vehicle treatment group. To investigate the effect of 0.5% CMC-Na intragastrically per day for one week on ischemia reperfusion injury.

Figure S3 Schematic diagram for ischemia hemisphere.
