# Supplementary figures and images for: Kaempferol Protects Against Cerebral Ischemia Reperfusion Injury Through Intervening Oxidative and Inflammatory Stress Induced Apoptosis
Source: Front Pharmacol. 2020 Apr 15;11:424. doi: 10.3389/fphar.2020.00424 (PMC7174640; doi:10.3389/fphar.2020.00424)

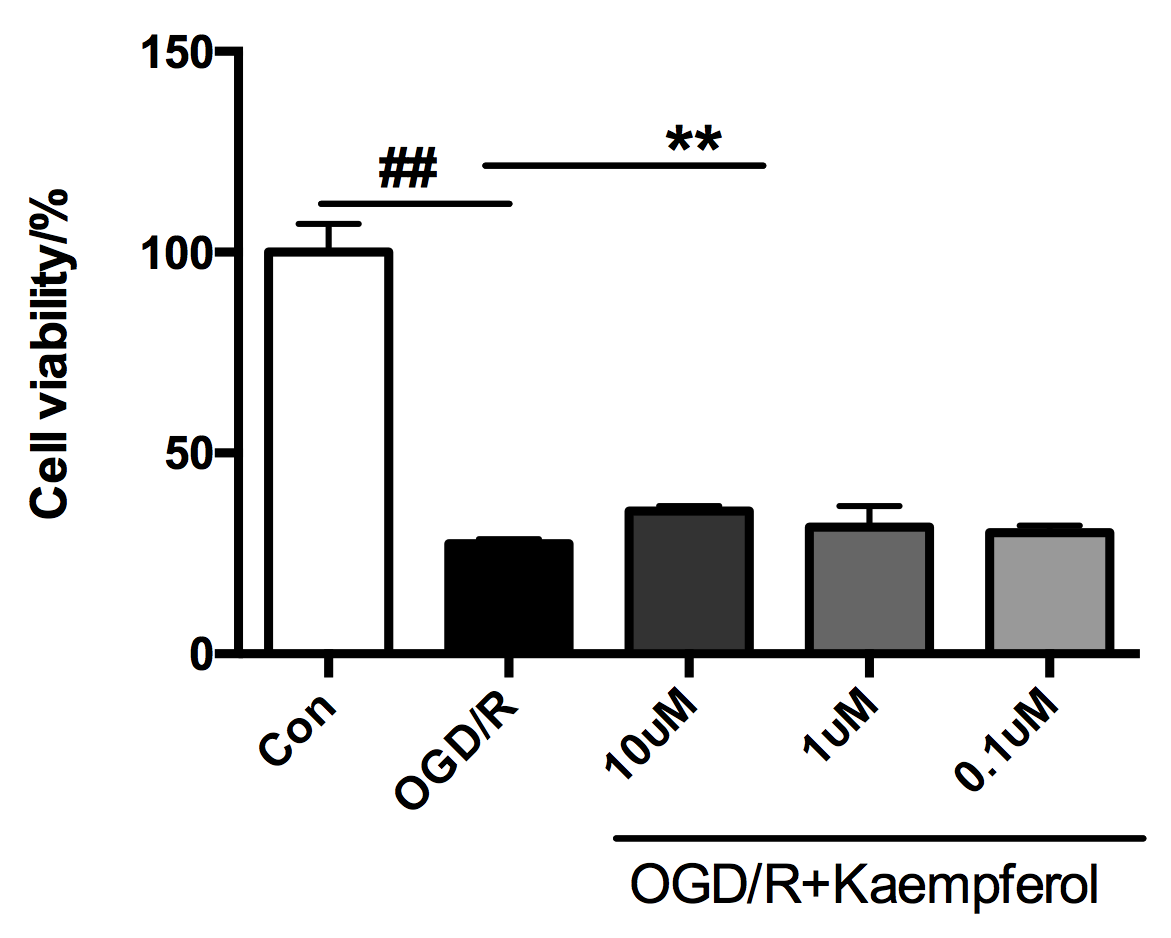

Supplement: Figure S1 — Kaempferol increased the cell viability under OGD/R. ##P < 0.01, Con group vs. OGD/R group; **P < 0.01, OGD/R group vs. OGD/R + Kaempferol 10μM treatment group. [file Image_1.tiff]

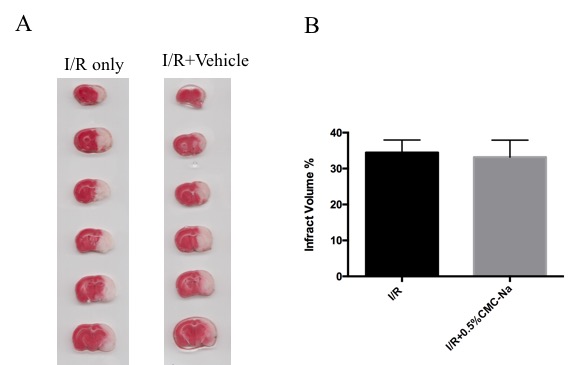

Supplement: Figure S2 — The infract volume in I/R only group and I/R+vehicle treatment group. To investigate the effect of 0.5% CMC-Na intragastrically per day for one week on ischemia reperfusion injury. [file Image_2.jpeg]

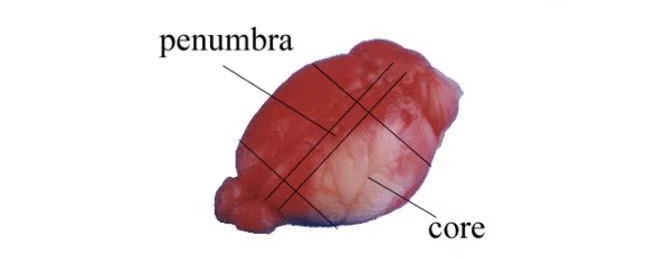

Supplement: Figure S3 — Schematic diagram for ischemia hemisphere. [file Image_3.jpeg]
